# An Acute Dose of Specific Grape and Apple Polyphenols Improves Endurance Performance: A Randomized, Crossover, Double-Blind versus Placebo Controlled Study

**DOI:** 10.3390/nu9080917

**Published:** 2017-08-22

**Authors:** Gaëlle Deley, Damien Guillemet, François-André Allaert, Nicolas Babault

**Affiliations:** 1 CAPS, U1093 INSERM, Université de Bourgogne-Franche-Comté, Faculté des Sciences du Sport, F-21000 Dijon, France; gaelle.deley@u-bourgogne.fr; 2 Centre d’Expertise de la Performance, U1093 INSERM, Université de Bourgogne-Franche-Comté, Faculté des Sciences du Sport, F-21000 Dijon, France; 3 Nexira, 129 Chemin de Croiset, F-76000 Rouen, France; d.guillemet@nexira.com; 4 CEN Nutriment, Impasse Françoise Dolto, F-21000 Dijon, France; allaert@groupecen.com

**Keywords:** maximal exertion, aerobic, cycling

## Abstract

Polyphenols are thought to be an interesting ergogenic aid for exercise and recovery. However, most studies regarding the effects of polyphenols investigated several days of supplementations. The present work aimed to study the effects of an acute intake of grape and apple polyphenols on the capacity to maintain intense exercise, here named endurance performance. Forty-eight physically active men (31 ± 6 years) were included in this study. During the two testing sessions, volunteers completed an endurance test at a high percentage of their maximal aerobic power and time to exhaustion was measured. Respiratory and pain parameters were also monitored. The preceding evening and 1 h before testing, volunteers had to absorb either 500 mg of polyphenols or placebo according to randomization. In comparison with the placebo, the mean duration of the maximal endurance test was significantly increased with polyphenols (+9.7% ± 6.0%, *p* < 0.05). The maximal perceived exertion was reached later with polyphenols (+12.8% ± 6.8%, *p* < 0.05). Practically, the present study showed the beneficial effects of grape and apple polyphenols for athletes looking for endurance performance improvements. The specifically designed profile of polyphenols appeared to enhance the capacity to maintain intensive efforts and delay perceived exertion.

## 1. Introduction

Endurance performance during high intensive exercises is mainly determined by the capacity of the aerobic metabolism [1]. It generally induces muscle fatigue, defined as the reversible decline in skeletal muscle contractile function [2]. Fatigue is multifactorial and is often associated with many physiological parameters including reduced neural input and disruptive metabolic changes in skeletal muscles such as lactic acidosis and the production of oxidative free radicals [2]. Moreover, it could lead to oxidative stress as a result of an imbalance between reactive oxygen species (ROS) production and intrinsic antioxidant defense [3].

To alleviate oxidative stress, some ergogenic strategies have been tested. Numerous studies have reported that different types of supplementation such as polyphenols were of interest to protect against these mechanisms [4,5,6]. Indeed, although some studies demonstrated no or harmful effects [7,8], most studies observed the positive effects of antioxidants on oxidative stress or performance [9,10,11]. More particularly, polyphenols, have great antioxidant capabilities and protective effects [12,13]. In addition, polyphenols increase the synthesis and bioavailability of nitric oxide (NO) [14,15] which is well known as the most important mediator of vasodilation [16]. NO also plays an important role in many functions such as blood flow, mitochondrial respiration and platelet function [17]. As a consequence, the beneficial effects of NO have been demonstrated on muscle strength [18,19], but also during recovery following intensive efforts [20].

To date, most of the studies exploring the effects of polyphenols investigated several days or weeks of supplementation on vascular, blood parameters (blood pressure, NO concentration, oxidative stress markers) or endurance performance [4,21]. For instance, Trinity et al. [22] observed no alteration in the cycling time-trial after seven days of polyphenol dietary supplementation. However, conflicting results are often obtained [19]. According to a recent review [23], depending on the type of polyphenols, chronic consumption has potentially detrimental to promisingly beneficial effects. Only few studies have investigated the effects of a single intake on immediate performance and recovery capacity [5,10,24,25,26]. Therefore, the present work aimed to study the effects of an acute intake of a specific profile of polyphenols from grape and apple on physical performance. More specifically, performance in the present study referred to a high-intensity cycling exercise until exhaustion revealing the capacity to maintain a constant strong effort hereafter named endurance. We therefore hypothesized that an acute supplementation of polyphenols would increase the time to exhaustion during a high-intensity cycling exercise.

## 2. Materials and Methods

### 2.1. Experimental Overview

The primary objective of this randomized, crossover, double-blind and controlled study was to evaluate the effect of an acute intake of polyphenols supplement versus Placebo (maltodextrin) on endurance measured during a cycling test until exhaustion. Participants were tested on two separate occasions with a constant-load ergocycle. Before test sessions, participants were asked to absorb either two capsules of 250 mg of grape and apple polyphenols or two capsules of placebo according to randomization. The main parameter, i.e., time to exhaustion during each session, was tested using a Student’s *t*-test.

### 2.2. Participants

**Figure 1:**
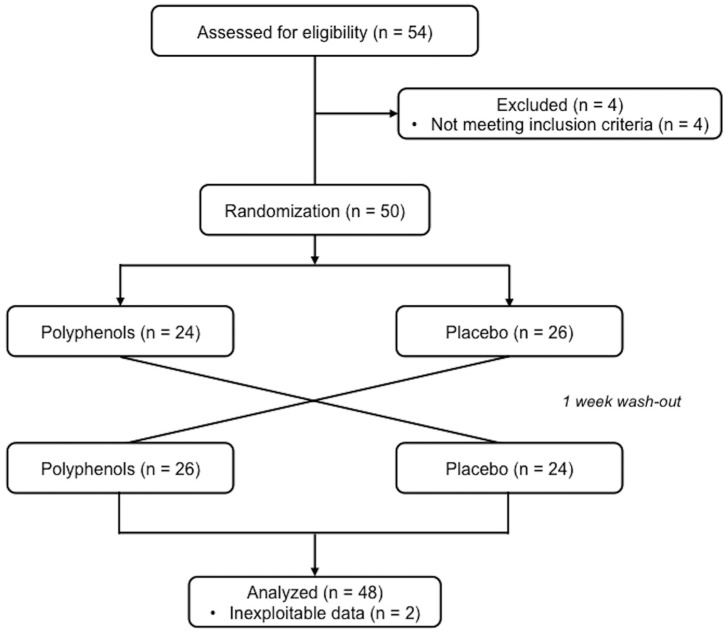
CONSORT flowchart.

A total of 48 healthy physically active males (exercising from 3 to 6 h a week) were recruited for the study (Figure 1). Volunteers with more than 6 h training a week, regularly trained in aerobic activities, who were asthmatic, smokers or under medicinal drugs, dietary supplement, sports drink, special dietary food or functional food, of any kind, liable or presented as liable to enhance physical performances, were excluded from the study. Throughout the study, volunteers maintained their usual training routine and diet. All gave their written informed consent after being told about the experimental protocol. The study was conducted in accordance with the Helsinki Declaration, was approved by the local ethics committee (CCP Est I: 2011/57) and was registered at ClinialTrials.gov (NCT03214276). Volunteers’ characteristics are presented on Table 1. This sample size was calculated a priori using Nquery Advisor (version 6.01) software based on the primary criterion and allowing for a power of more than 90%.

### 2.3. Experimental Procedure

After inclusion, participants came to the laboratory for three tests performed on a CycleOps 400 PRO equipped with PowerTap power meters (CycleOps, Madison, WI, USA) that allowed constant power output independently of pedalling rate. During the first visit, maximal aerobic power was determined during an incremental cycling test. Characteristics of the test were determined individually according to the equation of Hansen et al. [27] in order to have a test lasting between 8 and 15 min. Briefly, participants started at an intensity ranging from 45 to 60 W during 180 s followed by increments of 20 to 30 W every 60 s. The test was interrupted when participants were unable to maintain the requested cycling rate and the last power value maintained at least 30 s was considered as the maximal aerobic power. It was used as the reference during the two other test sessions. During this test, participants were asked to remain seated all the time and to keep a constant pedalling rate of 80 revolutions per minute. Saddle and handlebar settings were individually adjusted and used during the other test sessions. Heart rate was measured at rest, during the test and during 3 min after the end of the test (Polar, Polar Electro Oy, Kempele, Finland).

At least two days after the incremental test, participants underwent two constant-load exercises at an intensity corresponding to 70% of their maximal aerobic power until exhaustion. Pedalling rate was kept constant during the whole test and during each session. Also, participants were regularly encouraged by the experimenter using standardized sentences and timing. Participants were blinded for the duration of the test. The main parameter tested was the time to exhaustion, measured in seconds. Heart rate, blood pressure, ventilation, and gas exchanges (Cosmed K4b2, Cosmed, Rome, Italy) were also measured at rest, during the test and during five minutes after the end of the test. Beat by beat heart rate as well as breath by breath ventilation and VO_2_ were averaged every five seconds throughout the test. The average values over the last 30 s of the tests were considered as maximal values. Half-recovery times for heart rate and VO_2_ (i.e., the time necessary to obtain half the value measured at exercise end) were also calculated. Every 4 min during the all-out test, Borg scale was used to determine participants’ perceived exertion [28]. Muscle pain was finally evaluated 48 h after each experimental session using a numerical seven-point scale.

The two endurance tests were separated by at least 7 days (washout period) and were performed in the morning at the same hour, i.e., 2 h after a standardized breakfast composed of 125 mL orange juice, 80 g of wholemeal bread, 20 g of butter and 20 g of jelly. The preceding evening and one hour before the endurance test, participants were asked to absorb either two capsules of 250 mg of polyphenols (Vinitrox™) or two capsules of placebo (maltodextrin) according to the order defined by the randomization (similar appearance and flavour). Because polyphenols are partiallydirectly bioavailable [29] but also later after gut microbiota and liver metabolization [30], two intakes have been imposed (one hour prior exercise andthe evening before, respectively). Participants receiving polyphenols in the first session received placebo in the second and reciprocally. Both the participants and the experimenters were blinded from the randomization. Except the standardised breakfast, diet was not controlled. Nevertheless, participants were instructed to have an almost similar food intake the day before tests.

Vinitrox™, supplied by Nexira (France) is a combination of specific profile polyphenols from grape and apple. Vinitrox™ is a purified extract with low lipids, fibers and proteins content (respectively 0.5%, 1.4% and 5.5%) and more than 60% of total polyphenols (Folin method—expressed as gallic acid equivalent) with antioxidant properties demonstrated by ORAC value (12,000 μMtrolox equivalent per gram of ingredient). The main polyphenol classes are proanthocyanidins (as catechins, B2 dimer), phenolic acids (as chlorogenic acids, gallic acids) and anthocyanins (as malvidin-3-glucoside). The principal polyphenol group is monomeric and oligomeric forms of flavanols (which include proanthocyanidins) with more than 10% (high-performance liquid chromatography). Preliminary unpublished observations performed on endothelial human cells demonstrated statistical significant improvements of NO synthesis by these polyphenols. They allowed polyphenols formulation optimisation to reach the highest synergetic effect on endothelial nitric oxide synthase activation (via serine 1177 phosphorylation). This formulation demonstrated the ROS protection effect via downregulation of peroxinitrite (unpublished observations on in vivo trained hamsters) and NO-dependent vasodilation activation (unpublished observations on ex vivo rings of aorta from rats).

### 2.4. Statistical Analyses

Quantitative variables were presented as mean values and standard deviation. Qualitative variables were expressed as frequencies and percentages. Means are compared using a Student’s *t*-test and percentages using Chi square test. Statistics were conducted using SAS software (Ver. 9.2, SAS institute, Inc., Cary, NC, USA). *p* < 0.05 was taken as the level of statistical significance.

## 3. Results

All recruited participants performed the entire protocol and no adverse event was reported.

### 3.1. Endurance Test

In comparison with the placebo, the present study revealed a significant increase (+9.7% ± 6.0%, *p* < 0.05) of the time to exhaustion during the endurance test with polyphenols (Table 2). As shown in Table 2, no significant differences were obtained for the maximal and mean heart rate, the maximal blood pressure, the maximal and mean VO_2_, and the maximal and mean ventilation between endurance tests performed with polyphenols or with placebo. In contrast, the maximal perceived exertion showed a significant difference, and was reached 2.7 min later (+12.8% ± 6.8%, *p* < 0.05) with polyphenols than with placebo.

### 3.2. Recovery

The VO_2_ half-recovery time was significantly longer in the polyphenol condition as compared with the placebo (+8.5 ± 11.4 s, *p* < 0.05). No other differences were noticed for the parameters registered after the end of the tests. Finally, muscle pain perception, evaluated 48 h after each experimental session, was not different between conditions (1.7 ± 0.9 and 1.5 ± 0.8 for polyphenols and placebo, respectively; *p* = 0.753).

## 4. Discussion

The main aim of the present study was to investigate the effects of an acute intake of a specific formulation of polyphenols from apple and grape on endurance capacity and recovery. The present study demonstrated that the intake of polyphenols prior to an endurance exercise increased the time to exhaustion and lengthened the time to onset of maximal perceived exertion as compared with the placebo. Taken together, these results suggested an increased endurance capacity with the acute intake of polyphenols.

The mean endurance test duration was ~25.5 min and ~28.0 min with the placebo and polyphenols, respectively. Such a duration may appear low when considering the power output (70% of the maximal aerobic power). This short duration could firstly be attributed to the initial test that used 1 min increments which could overestimate the maximal aerobic power. Secondly, volunteers were physically active and not specifically trained for cycling. The inherent variability of this population and of the measurements was counterbalanced by the large sample size tested here and the randomized, crossover design. Indeed, as indicated in a recent review [11], most studies considered small samples. The 48 participants of this randomized, crossover, double-blind study therefore strengthen our conclusions.

The increased time to exhaustion is concordant with some previously published studies [25]. In this last study, the authors registered significant increases in the running time to exhaustion with the acute intake of polyphenols as compared to the placebo [25]. In addition to some increases in the endurance aerobic performance, other authors revealed some improvements in anaerobic power with caffeine-based products [10]. However, the few studies that investigated the effects of the intake of acute polyphenols are often conflicting. Other authors [22,26] did not register any effect of polyphenols on the cycling time-trial performance in elite or well-trained cyclists. Such discrepancies could be ascribed to the training status (elite vs. amateur athletes), nature and duration of exercise or to the type of polyphenols used [23]. For example, Jowko et al. [24] concluded that an acute intake of green tea polyphenols was not efficient to attenuate exercise-induced oxidative stress while Morillas-Ruiz [5] detected some protective effects of polyphenols (mostly from fruits) against exercise-induced oxidative stress. Also polyphenols from cranberries and grapes increased artery flow-mediated dilation [26].

It is important to note that the increased time to exhaustion with polyphenols, observed here, is obtained with a delayed fatigability (as witnessed by the late rate of perceived exertion), but with similar physiological responses compared to the placebo. Indeed, the heart rate, blood pressure, VO_2_ and ventilation rate are similar between conditions. These results are concordant with previous findings [10]. 

Some studies attributed antioxidants’ and more particularly polyphenols’ effects to enhanced blood flow [26]. Although not tested here, several mechanisms might explain the present increase in endurance through an action on NO. Indeed, polyphenols (notably from grape sources) have great antioxidant capabilities [12,13] and increase the synthesis and bioavailability of NO [14,15], thus having the potential to delay fatigue. Additionally, polyphenols such as green tea or grape have been associated to improved endothelial function [31]. Based on in vivo and ex vivo preclinical unpublished observations with Vinitrox™, we could speculate that performance benefits might be due to the modulation of NO-dependent vasodilation with NO synthesis increase and protection. Previous studies demonstrated that this antioxidant effect might also contribute to improving NO effects by two main actions. Firstly, its lifespan protects against O_2_^−^ and, secondly, it prevents eNOS uncoupling, leading notably to a lower flow-mediated dilation of arterioles [32,33].

Previous studies demonstrated that the primary mechanism of NO is the increase in muscle perfusion through a direct vasodilator action on vascular smooth muscle cells and an inhibition of adrenergic vasoconstriction [34,35]. This muscular hyperemia might induce an increased oxygen supply to muscle cells, as well as higher nutrient supply and metabolite product removal, which would allow an enhanced aerobic metabolism [25]. Another mechanism may lie in the action of NO on the glucose metabolism of the muscle, in particular through an increased muscle uptake. In addition, the endogenous production of NO on the sarcoplasmic reticulum Ca^2+^ is likely to improve muscle contractile performance [36,37].

The second aim of the present experiment was to assess the effects of polyphenol supplementation on exercise recovery. Indeed, an NO production increase is supposed to enhance oxygen and nutrient delivery to active muscles, thus improving tolerance to physical exercise and recovery mechanisms [16]. Contrarily, our results revealed a significant lengthening of the time of half-recovery of the VO_2_ under the polyphenol condition. Therefore, the lengthening of the recovery that could reflect the existence of an oxygen debt might be surprising when considering NO effects. However, it is in agreement with the longer duration of the endurance tests. Indeed, the duration of the O_2_ debt has been shown to be directly related to the exercise duration [38]. The longer recovery could therefore be primarily attributed to the longer-endurance exercise (obtained with polyphenols ingestion) rather than physiological mechanisms related to polyphenol ingestion and potential vasodilation effects. However, additional measurements are necessary to verify this speculative statement. Our result might be of great interest, particularly for people wishing to lose weight since the lengthening of the O_2_ debt is associated with increased energy expenditure, and more specifically lipids oxidation [38,39].

Lastly, the absence of any muscle pain two days after exercise in both conditions indicates that this acute polyphenol supplementation allowed participants to perform longer exercises without further adverse effects. This is of particular interest for athletes training regularly since one of the limiting factors of training is often muscular pain resulting from effort.

In conclusion, the present randomized, crossover, double-blind and controlled study demonstrated that the acute supplementation of polyphenols in healthy, physically active males allowed significant increases in endurance performance (hereby the capacity to maintain a strong effort) with greater energy expenditure as demonstrated by the lengthening of time to exhaustion and time to maximal perceived exertion. Also, the main cardiovascular and respiratory measured parameters showed no significant differences between conditions. A similar observation was obtained on muscle pain two days after exercise. These results indicate that performance improvements, as a result of acute polyphenol intake, have been obtained under safe conditions and without additional pain. In contrast with some previous studies, the present conclusions were made on an almost large sample size and reinforce the positive effects of polyphenols on cycling endurance. An interesting perspective of this work would be to control diet during the duration of the experiment and to quantify specific biomarkers in order to better understand the mechanisms behind the present results.

## Figures and Tables

**Table 1:** Participants’ characteristics after inclusion.

Characteristics	Mean Values
Age (years)	31.0 ± 6.0
Height (cm)	181.2 ± 6.4
Weight (kg)	77.3 ± 9.3
Weekly activity (h/wk)	3.9 ± 1.0
BMI (kg/m^2^)	23.5 ± 2.2
Resting HR (bpm)	62.4 ± 9.3
Resting VO_2_(mL/min/kg)	4.6 ± 1.3
Resting SBP (mm Hg)	129.0 ± 8.3
Resting DBP (mm Hg)	75.7 ± 6.7
Maximal aerobic power (Watts)	294.4 ± 52.4

Values are means ± standard deviation. BMI: body mass index, HR: heart rate, VO_2_: oxygen consumption, SBP: systolic blood pressure, DBP: diastolic blood pressure.

**Table 2:** Parameters recorded during and after endurance tests in polyphenols and placebo conditions.

	Polyphenols	Placebo	*p* Value
**Time to exhaustion (s)**	1680.2 ± 779.2	1531.5 ± 643.5	0.032
**Mean HR (bpm)**	166.9 ± 12.0	166.7 ± 12.9	0.917
**Mean VO_2_ (mL/min/kg)**	40.5 ± 5.2	41.5 ± 7.0	0.251
**Mean VE (L/min)**	94.0 ± 15.9	95.6 ± 16.6	0.313
**Maximal HR (bpm)**	178.2 ± 12.0	178.3 ± 12.9	0.952
**Maximal VO_2_ (mL/min/kg)**	42.4 ± 5.3	43.1 ± 5.3	0.236
**Maximal VE (L/min)**	114.8 ± 22.6	117.7 ± 22.1	0.226
**Maximal systolic BP (mm Hg)**	143.2 ± 14.3	144.9 ± 17.4	0.489
**Maximal diastolic BP (mm Hg)**	78.1 ± 6.3	77.8 ± 8.3	0.835
**Time to reach maximal perceived exertion (s)**	1434 ± 594	1272 ± 540	0.005
**Half-recovery time for VO_2_ (s)**	60.9 ± 21.1	52.4 ± 15.2	0.014
**Half-recovery time for HR (s)**	194.0 ± 95.6	191.5 ± 99.4	0.888
**Muscle pain**	1.7 ± 0.9	1.5 ± 0.8	0.753

Values are means ± standard deviation. HR: heart rate, VO_2_: oxygen consumption, VE: ventilation.
